# Organic Diets and Children’s Health

**DOI:** 10.1289/ehp.114-a210b

**Published:** 2006-04

**Authors:** Alex Avery

**Affiliations:** Center for Global Food Issues, Hudson Institute, Churchville, Virginia, E-mail: aavery@cgfi.org

In their article “Organic Diets Significantly Lower Children’s Dietary Exposure to Organophosphorus Pesticides,” Lu et al. (2006) used language that is likely to be misused by organic food marketers to promote high-priced foods and could discourage lower-income parents from providing their children with a diet rich in fruits and vegetables.

Regarding their findings that children’s median urinary concentrations of two organophosphorus (OP) pesticides dropped to nondetectable levels within 24–48 hr after switching to an organic diet, Lu et al. (2006) state in the “Abstract” that “an organic diet provides a dramatic and immediate protective effect against exposures” and that these results provide “evidence of the effectiveness of this intervention.” Later in their article, they admit that they “did not collect health outcome data in this study,” but they claim that

It is intuitive to assume that children whose diets consist of organic food items would have a lower probability of neurologic health risks, a common toxicologic mechanism of the OP pesticide class.

This statement, in particular, seems tailor-made to mislead consumers into believing that organic foods will protect against actual neurologic health risks. A previous article by one of the coauthors presenting similar findings also contains potentially misleading wording (Curl et al. 2003). In the “Abstract,” Curl et al. (2003) stated that

Consumption of organic fruits, vegetables, and juice can reduce children’s exposure levels from above to below the U.S. Environmental Protection Agency’s [EPA] current guidelines, thereby shifting exposures from a range of uncertain risk to a range of negligible risk.

Collectively, the wording of both papers strongly implies to consumers and nonspecialists that consuming organic foods reduces likely or actual harm caused by residues of OP pesticides. However, evidence of harm from exposure to the low levels of OP pesticide residues in food is completely lacking in children or adults. Although there is some evidence from animal experiments that *in utero* exposures to OP pesticides at high enough doses can cause neurodevelopmental effects (Eskenazi et al. 1999), the doses at which such effects were seen were at least three orders of magnitude higher than those consumed as food residues by the children in these two studies (Curl et al. 2003; Lu et al. 2006).

Recent measurements of OP metabolites in the U.S. population by two of the authors of the Lu et al. study (Barr et al. 2005) allowed estimations of doses at the 95th and 50th percentiles of the population for chlorpyrifos (the OP exposure closest to the U.S. EPA reference dose). Barr et al. (2005) estimated that at the 95th percentile, children still consumed less than one-half of the U.S. EPA’s chronic population-adjusted dose (cPAD), confirming the exposure estimates used in the risk assessment of the Health Effects Division at the U.S. EPA in 2000 (Barr et al. 2005). The cPAD for chlorpyrifos is 1/1,000th of the no observable adverse effect level (NOAEL) in dogs and rats. Thus, children in the 95th percentile consumed < 1/2,000 of the NOAEL, and the median exposure in children was 1/5,000 of the NOAEL.

If it is appropriate to intuitively assume that organic foods pose a lower probability of risk to children, is it not also appropriate to clearly state that all of the risks discussed in these articles are negligible, given that they are tiny fractions of the NOAEL in the most sensitive animal species? It seems that the language chosen by these authors was not appropriate. Already, organic food marketing interests are using these articles as “proof” that organic food is better for you (Organic Consumers Association 2003); Lu et al.’s article is even posted on the Organic Consumers Association website (Organic Consumers Association 2005).

In the future, those of us who communicate with the public on food safety issues should choose our words carefully, not make claims that go beyond the scope of the research, and take the time to accurately place the level of risks being discussed within the context of what is known from animal studies.
